# Retraction Note: Air-stable superparamagnetic metal nanoparticles entrapped in graphene oxide matrix

**DOI:** 10.1038/s41467-020-19968-3

**Published:** 2020-11-25

**Authors:** Jiří Tuček, Zdeněk Sofer, Daniel Bouša, Martin Pumera, Kateřina Holá, Aneta Malá, Kateřina Poláková, Markéta Havrdová, Klára Čépe, Ondřej Tomanec, Radek Zbořil

**Affiliations:** 1 Department of Experimental Physics and Department of Physical Chemistry, Regional Centre of Advanced Technologies and Materials, Faculty of Science, Palacky University in Olomouc, Slechtitelu 27, 783 71 Olomouc, Czech Republic; 2 Department of Inorganic Chemistry, University of Chemistry and Technology Prague, Technická 5, 166 28, Prague, 6 Czech Republic; 3 Division of Chemistry & Biological Chemistry, School of Physical and Mathematical Sciences, Nanyang Technological University, 637371 Singapore, Singapore; 4 Institute of Scientific Instruments, The Czech Academy of Sciences, Kralovopolska 147, 612 64 Brno, Czech Republic

Retraction of: *Nature Communications* 10.1038/ncomms12879, published online 15 September 2016.

The authors are retracting this Article because two figures with Mössbauer spectra have raised concerns. For Supplementary Fig. 6 and related discussion on the stability of the superparamagnetic iron nanoparticles, we no longer had the raw data and are thus unable to verify the claims in the published paper. Additionally, due to very low Mössbauer effect of superparamagnetic iron nanoparticles embedded in graphene oxide matrix and related low statistical quality of raw Mössbauer data, there is uncertainty in values of Mössbauer parameters derived from Figure 2, and we, the authors, therefore wish to retract this Article.

